# Waiting, Thinking, and Feeling: Variations in the Perception of Time During Silence

**DOI:** 10.3389/fpsyg.2020.00602

**Published:** 2020-04-02

**Authors:** Eric Pfeifer, Marc Wittmann

**Affiliations:** ^1^Catholic University of Applied Sciences, Freiburg, Germany; ^2^Institute for Frontier Areas of Psychology and Mental Health, Freiburg, Germany

**Keywords:** relaxation, silence, time perception, waiting, music therapy, logotherapy, psychotherapy

## Abstract

Research on the perception of silence has led to insights regarding its positive effects on individuals. We conducted a series of studies during which individuals were exposed to several minutes of silence in different contexts. Participants were introduced to different social and environmental settings, either in a seminar room at a university or in a city garden, alone or in a group. Instructions across studies varied, as participants were exposed to real waiting situations, were asked to just think and to explicitly experience the time interval without further instructions or following a session of Depth Relaxation Music Therapy (DRMT)/Hypnomusictherapy (HMT). Silence was judged to significantly increase relaxation, improve mood states, and alter the perception of time and the orientation toward the present moment. We controlled for influences of trait variables, such as impulsivity, mindfulness, daydreaming, and time perspective. Findings empirically demonstrate that exposure to silence can be effective in therapeutic and educational contexts to promote relaxation and well-being.

## Introduction

“I believe silence is the new luxury. Silence is more exclusive and long lasting than other luxuries” (Kagge, 2018). Erling Kagge, author of the recently-published book “Silence: In the Age of Noise,” writes about silence and noise in the context of the current *zeitgeist*. Especially urban living seems to suffer from a loss of the luxury good silence due to an increasing amount of everyday noise. Governments and communities identify this development as a specific challenge for populations, the environment, and nature. They call for innovative approaches and provide research funds to meet this challenge (e.g., the German Federal Ministry of Education and Research, 2016). Noise, among other factors, could be responsible for the confirmed higher risk of individuals living in urban areas to develop mental disorders, like schizophrenia, depression, and anxiety, as compared to residents in rural areas (Lederbogen et al., 2011; Lederbogen and Meyer-Lindenberg, 2016). The interdependence between health and noise is evident in healthcare institutions. Studies show that noise in hospitals negatively affects the patient’s recovery (Mazer, 2010), the development of preterm infants in neonatal intensive-care units (NICU) (Gilad and Arnon, 2010), and patient care and causes stress reactions among patients in emergency departments (ED) (Short et al., 2010). In contrast, silence was found to lower the diastolic blood pressure, heart rate, and breathing rate and to decrease cortisol levels (Bernardi et al., 2006; Trappe and Voit, 2016). Listening to slow or meditative music led to even greater relaxation if combined with randomly-inserted pauses (Bernardi et al., 2006).

Silence may not be a prominent topic in current health-related research, but there is evidence of growing interest. For example, the exploration of silence was listed as one of the newly developing main research topics in present-day music therapy (Oberegelsbacher and Timmermann, 2012). Sitting in silence is an important aspect of mindfulness and meditation practices (Black et al., 2009), and silent meditation leads to alterations in the senses of time, space, and self (Berkovich-Ohana et al., 2013; Thönes and Wittmann, 2016). In a series of studies, Wilson et al. (2014) found out that students did not enjoy “just thinking” in silence alone in a room and that “[…] being alone with their own thoughts for 15 min was apparently so aversive that it drove many participants to self-administer an electric shock that they had earlier said they would pay to avoid.” Buttrick et al. (2018) repeated the study by Wilson et al. and similarly concluded that participants preferred to occupy themselves with activities, such as watching TV or reading a book, rather than “just thinking” or turning one’s attention inward during silence while sitting alone in a room. Fox et al. (2014) published a critical review of Wilson et al.’s findings and stated that spending time with our own unstructured thoughts (mind-wandering, daydreaming) may not be as enjoyable or entertaining as structured activities, but may “increase our overall sense of well-being and life satisfaction.” According to this reanalysis of Wilson et al.’s data, there is not actually much support for the notion that participants found the period of “just thinking” to be unpleasant. On average, Fox et al. showed that the response curve is normally distributed with average empirical values close to the means of the scales. It is only relative to a situation of being active with a task or entertainment that “just thinking” is less pleasurable.

Recent studies indicate that “thinking for pleasure” does not come easily; it may even be cognitively demanding, although simple cognitive aids and the right conditions can make it easier and even enjoyable (Westgate et al., 2017; Wilson et al., 2019). Motivation – in terms of motivating subjects to try to enjoy their own thoughts – was also judged to be an important catalyst in making an activity, such as “just thinking” or “thinking for pleasure,” enjoyable (Alahmadi et al., 2017). In contrast to Wilson et al.’s above-mentioned findings, Nguyen et al. (2017) stated that 15 min of solitude, sitting on a comfortable chair in silence and alone in a room, effectively reduces arousal and fosters self-regulation. Solitude helped subjects calm down, become quiet, and to regulate their affective states. Silence and related concepts (e.g., mind-wandering, daydreaming, just thinking, thinking for pleasure, meditation, mindfulness, empty time) are being discussed in an increasing number of studies. We conducted a series of studies that focused on silence in various settings (e.g., in a city garden, in a university lecture room) and situations (e.g., as part of a relaxation session, in a waiting situation). Herein we discuss our main outcomes in reference to health, well-being, and therapeutic and educational settings and theories. We structured our review by creating content-related clusters framing the presentation of the individual studies.

(1) Waiting-room study (Jokic et al., 2018).
(2) “Just think” study (Pfeifer et al., 2019c).
(3) Depth relaxation studies (Pfeifer et al., 2016, 2019a).
(4) “Pure” silence study (Pfeifer et al., 2019b).

## Review

### Waiting-Room Study (Study 1)

The study by Jokic et al. (2018) assessed the participating students’ experience of an empty time interval of 7:30 min duration as part of a real waiting situation. Relations between the students’ experience of time while waiting and facets of affective well-being, individual impulsivity traits, and time perspectives were of specific interest to us. An earlier study we had conducted was the first attempt to capture a form of waiting time in which we had participants retrospectively judge the duration of a 5 min, resting-state period (reference) by measuring the psychophysiological indices of heart rate and breathing rate (Wittmann et al., 2017). More impulsive individuals relatively overestimated this resting state period.

We then conducted a real waiting study where subjects were given a fictitious explanation of why they had to wait alone in a room. 82 students (51 women, 31 men; mean age: 22.5 years; *SD* = 3.7; range: 18–36) took part. The study was conducted in Thessaloniki, Greece; the majority of the participants were students from local universities. Half of the participants were asked to complete trait-related questionnaires before they went into the waiting room. Afterward, they reported their experience by answering state-related questionnaires. The other half of the students began with the waiting-room situation, then answered the state-related questionnaires, and finally completed the trait-related questionnaires.

Each student spent exactly 7:30 min of silent waiting time (the duration was unknown to the subjects) alone in a very basic, closed room with a desk and a chair. We also asked students to hand over all their belongings, such as mobile devices and bags which might have contained reading materials, before entering the room. In the room, the subjects were told to simply wait for the researcher to come back, as she had to set up the computer in another room. This was the fictitious explanation for the real waiting time.

The Zimbardo Time Perspective Inventory (ZTPI) (Zimbardo and Boyd, 1999) and the Barratt Impulsiveness Scale (BIS-11) (Barratt et al., 1999) were used as trait scales. We had already used the state scales on the subjective time, self, and space (STSS) to assess states of consciousness during silence after depth relaxation (Pfeifer et al., 2016). A non-verbal, pictorial-assessment technique, the Self-Assessment-Manikin (SAM) (Bradley and Lang, 1994), was included to determine the emotions the participants experienced most of the time while waiting alone in the room. A visual analog scale (VAS) concerning felt relaxation was also administered.

Correlational analyses showed that students with more positive emotions (SAM), lower arousal scores, and greater experienced relaxation (VAS) estimated the duration of the waiting period as relatively shorter. The waiting period was over-estimated, and the passage of time was felt to pass slower when individuals felt more irritated and aroused. Correlations with trait variables showed that the higher the scores for self-rated impulsivity and fatalistic and hedonistic present orientation, the less relaxed students were and the greater their overestimation of the duration of the waiting time. Subsequent path analyses revealed an integrative view of the interconnections among these variables. The level of relaxation has an indirect (mediating) effect on subjective time: more impulsive people feel less relaxed, overestimate duration, and experience a slower passage of time.

Interrelations between emotional states and time judgments (Wittmann, 2009; Lambrechts et al., 2011; Schäfer et al., 2013; Wackermann et al., 2014; Droit-Volet et al., 2015), as well as between impulsivity traits, present and future orientation, and time perception (Baumann and Odum, 2012; Mueller et al., 2014; Wittmann et al., 2017), have been reported in previous studies. Higher arousal states and an impulsive present orientation lead to relative overestimations of duration. Our study may be the first that systematically assessed subjective time in a real waiting situation in association with affective reactions and individual differences.

### “Just Think” (Study 2)

“Just thinking,” or engaging oneself with one’s own thoughts alone in a room, was the core element of silence in this study (Pfeifer et al., 2019c). Wilson et al. (2014) had claimed that students did not enjoy a period of silence lasting between 6 and 15 min spent alone in a room with nothing to do but occupy themselves with their own thoughts. Some individual participants even felt this situation to be so unpleasant that they preferred to self-administer an electric shock rather than spend their time “just thinking” while being alone with their own thoughts (see the critical assessment of these claims in the introduction).

We conducted a variation of this investigation relying on the principle study design applied by Wilson et al. (2014), who did not assess time in their study, although it is an essential experience related to waiting. The employed state inventories referred to the conscious dimensions of subjective time, space, and self (STSS), which have proven suitable for assessing longer time intervals, such as a real waiting-time situation (Jokic et al., 2018), a silence after Depth Relaxation Music Therapy (DRMT)/Hypnomusictherapy (HMT) (Pfeifer et al., 2016), and watching a dance performance (Deinzer et al., 2017). Two visual analog scales were used to assess relaxation and boredom, and the Self-Assessment-Manikin (SAM) (Bradley and Lang, 1994) scale was implemented to assess emotional reactions. The Zimbardo Time Perspective Inventory (ZTPI) (Zimbardo and Boyd, 1999) and the Barratt Impulsiveness Scale (BIS-11) (Barratt et al., 1999) were used as trait questionnaires. Sixty four undergraduate and graduate students (51 women, 13 men; mean age: 26.7 years; *SD* = 6.9; range: 19–52) studying “Inclusive Education,” “Education,” “Social Work/Social Education” or other programs at the Catholic University of Applied Sciences in Freiburg took part in our investigation.

The sessions were held in a seminar room at the Catholic University of Applied Sciences Freiburg. It was a functional room with plain white walls (no posters, shelves, photographs, etc.). The participants were asked to seat themselves with their backs to the window facing the door. After a short introduction (providing relevant information, informed consent, students handing over their belongings, such as cell phones, tablets, etc.), the students filled out the questionnaires and were then asked to spend a period of silent time on their own. The exact instruction was: “Please spend the following time occupying yourself with your own thoughts and please stay seated and awake.” The period of silence lasted 6:30 min, but the time span was unknown to the participants. The researcher returned after the interval had elapsed and asked each participant to fill out the set of state questionnaires.

Participants felt significantly more relaxed (VAS), in a better mood (SAM), and less aroused (SAM) after vs. before the 6:30 min of silence alone in a room. Boredom was hardly felt (an average of 10.4 on the 100 mm VAS), participants were on average quite focused on the present moment (53.1%; 19.6% on the past, 27.3% on the future), and time seemed to pass comparably fast for them (an average of 77 on the 100 mm VAS). Time was not felt very intensely (an average of 32.1 on the 100 mm VAS), whereas the self was felt quite intensely (an average of 5.0 on a scale between 0 and 6). The instruction to wait and occupy oneself with one’s own thoughts apparently led to more self-awareness, but in an emotionally positive way.

We emphasize that we did not tell the students beforehand how long the silent period would last. Such situations of uncertainty are typically experienced as irritating (Zakay, 2015). In the study by Wilson et al. (2014), the participants were given specific or approximate information about the waiting period of 10–15 min. Although Wilson et al. claimed that their study participants felt quite irritated while waiting, they actually felt less pleasant only relative to situations in which they were actively engaged. The re-analysis of the Wilson et al. (2014) study by Fox et al. (2014) showed little support for the notion that participants found the period of “just thinking” to be aversive. Subjects rated the experience of engaging themselves with their own thoughts with mean values around 50 mm on the VAS. These results and our own indicate that people on average do not feel uncomfortable when just thinking.

### Depth Relaxation (Studies 3 and 4)

This section provides details on two studies (Pfeifer et al., 2016, 2019a) in which a period of silence followed a DRMT/HMT session or a control condition. The main difference between these two studies lies in the setting where they took place: study three (Pfeifer et al., 2016) was performed indoors and study four (Pfeifer et al., 2019a) outdoors in a city garden.

DRMT/HMT as the experimental intervention in our two studies was developed by Hans-Helmut Decker-Voigt (2007, 2009) and has been influenced by various techniques and approaches, such as expressive art therapy, Gestalt theory, Milton Erikson’s hypnotherapy, autogenic training, psychoanalytic and humanistic psychology, and guided imagery. DRMT/HMT is a therapeutic method to facilitate relaxation. It can be applied in various therapeutic contexts, supports the general aims of music therapy as framed by various professional associations (World Federation of Music Therapy (WFMT), 2017; American Music Therapy Association, 2019; German Association of Music Therapy (DMtG), 2019), and proceeds along seven steps or “building blocks.” The building blocks are, for example: “sensitization to the feeling of ‘comfort’ in the body,” “sensitization to feelings, mental images, thoughts,” and “sensitization to the auditory perception of music.” A time limit from 3 to 8 min is suggested for the receptive music phase in DRMT/HMT. Longer periods could create difficulties in the participants’ reorientation. Instead of “normal” music, we used silence during the DRMT/HMT receptive music phase.

#### Depth Relaxation Indoors (Study 3)

Sixty students (45 women, 15 men; mean age: 22.9 years; *SD* = 2.6; range: 19–31) completed the study (Pfeifer et al., 2016). All were enrolled in undergraduate and graduate programs (“Inclusive Education,” “Social Work/Social Education,” etc.) at the Catholic University of Applied Sciences. The students were divided into five groups, three of which first received a 16 min session of DRMT/HMT followed by 6:30 min of silence (the intervention). One week later, these three groups had a 16 min seminar focusing on aspects of silence in therapy and counseling succeeded by 6:30 min of silence (the control condition). The other two groups also received both conditions, but in reverse order. The sessions took place in a seminar room at the Catholic University of Applied Sciences Freiburg and commenced with a brief instruction providing information on the procedure and other relevant details. Students were also asked to switch off any mobile devices and to take off their wrist watches.

The DRMT/HMT sessions were led by a professional music therapist (the first author of this article, E.P.) who accompanied the participants through the DRMT/HMT steps (“building blocks” I–V) by using speech to induce depth relaxation. All subjects were asked to remain in a seated position during the procedure while the therapist provided positive connotations (“All the thoughts circulating in your mind are allowed to do so…”) and suggested the participants make themselves comfortable by changing the positions of their feet, backs, heads, etc. After 16 min of DRMT/HMT, the therapist invited the students to focus their attention on the acoustic surrounding. The following 6:30 min period of silence was of unknown duration to the students. A subsequent short phase of re-orientation to the “here and now” (building block V) ended the session and initiated the final step, during which the participants filled out the questionnaires.

The students in the control condition were invited to participate in a group discussion and to share their experiences, ideas, and opinions concerning silence, its forms of occurrence and applications in health-related, therapeutic, and counseling settings. Case examples, the beneficial potentials of silence, risks, indications, and contraindications were discussed. The discussion was moderated by the same music therapist (E.P.), lasted 16 min, and was followed by a 6:30 min period of silence of unknown duration to the students. Afterward, the participants were asked to fill out the questionnaires while remaining seated.

The state scales we employed were the subjective time, self, space (STSS) and an additional VAS measuring relaxation. The trait scales we used were the BIS-11 (Barratt et al., 1999) on impulsiveness and the Freiburg Mindfulness Inventory (FMI) for the assessment of mindfulness (Walach et al., 2006).

Participants considered the 6:30 min of silence preceded by 16 min of DRMT/HMT significantly more relaxing than a silent period of the same duration following the seminar discussion. Students felt that the silence after DRMT/HMT vs. the seminar condition had lasted significantly longer, and their sense of space and time and future perspective was relatively reduced. The individuals’ levels of impulsiveness and mindfulness as trait variables did not affect these outcomes. The fact that silence after DRMT/HMT lasted subjectively longer than in the control condition is worth discussing. Typically, an overestimation of duration is associated with negative affect and increased arousal (Droit-Volet and Meck, 2007; Pollatos et al., 2014). Corresponding with other recent studies on the effects of mindfulness meditation which lead to an overestimation of the experienced duration in the range of milliseconds and seconds (Droit-Volet et al., 2015; Singh and Srinivasan, 2019), we showed that a similar overestimation in the range of minutes can be related to a more relaxed state of being in the present moment.

We conclude that silence after DRMT/HMT effectively promotes relaxation and well-being. The stronger present orientation after DRMT/HMT-induced silence indicates less rumination and circular reasoning, which are typical signs of irritation and anxiety about the past and future events. Previous studies have already highlighted the ability of music and music therapy to reduce anxiety (Walworth, 2003; Gimeno, 2010; Nguyen et al., 2010; Stegemann, 2013). Silence embedded in relaxation methods like DRMT/HMT could promote relaxation and effectively mitigate stress-related illnesses. In a group setting, this intervention can be cost effective in various health-related contexts, such as prevention programs.

#### Depth Relaxation Outdoors (Study 4)

Study 4 (Pfeifer et al., 2019a) had an almost identical design as study 3; it only differed in the environmental surrounding where the sessions were held and in the selection of some measuring instruments. The sessions in study 3 took place indoors (in a seminar room at the Catholic University of Applied Sciences). In study 4, 16 min of DRMT/HMT followed by 6:30 min of silence (the intervention) vs. 16 min seminar/group discussion on silence in therapy and counseling followed by 6:30 min of silence (the control condition) were performed in a city garden. All sessions were led by the same qualified music therapist (E.P.).

Eighty four participants (74 women, 10 men; mean age: 24.2 years; *SD* = 6.0; range: 20–58) completed the study. Most of the subjects were enrolled in health-related and/or social undergraduate or graduate programs (“Social Work/Social Education,” “Education,” “Inclusive Education,” “Nursing,” etc.) at the Catholic University of Applied Sciences in Freiburg. Some participants were visiting students studying music, music education, or psychology at other universities located in Freiburg. The students were divided into seven groups, four of which began with the control session (16 min of seminar/group discussion on silence in therapy and counseling followed by 6:30 min of silence) and received the experimental condition (16 min DRMT/HMT followed by 6:30 min of silence) 1 week later. The other three groups started with the experimental condition and had the control session 1 week later. The students were asked to switch off any mobile devices and to take off their wrist watches. They were asked to remain seated on the ground and awake during the whole process. The students did not know how long the period of silence would last.

The measuring instruments included the Daydreaming Frequency Scale (DDFS; Gutiérrez et al., 2019), the Multidimensional State Boredom Scale (MSBS; Fahlman et al., 2013), the scales on subjective time, self, space (STSS) (Pfeifer et al., 2016), and an additional VAS measuring relaxation.

Relaxation was significantly increased after silence in both conditions. On average, the level of relaxation was significantly higher after DRMT/HMT and silence as compared to before the intervention, as was the average increase in relaxation after the seminar and silence as compared to before. Being in the city garden led to increased relaxation, regardless of the intervention (DRMT/HMT vs. seminar discussion). The period of 6:30 min of silence after the DRMT/HMT condition was judged to pass significantly more slowly than after the seminar session. The students who were more relaxed during the experimental condition (DRMT/HMT) felt themselves more intensely, felt that time had passed more quickly, and experienced space to a lesser extent. On average, subjects after the DRMT/HMT condition felt that time had passed more slowly, but those individuals who were more relaxed as a result of the specific body-focused relaxation method felt that time had passed more quickly. Following DRMT/HMT, one subscale referring to the MSBS significantly correlated with relaxation: those participants who had had significantly higher scores on the boredom-arousal scale were less relaxed afterward. In the seminar condition, all MSBS subscales correlated with relaxation, indicating that the more bored individuals were before the seminar, the less relaxed they were after the period of 6:30 min of silence.

Previous studies (e.g., Ulrich, 1979, 1984; Berry et al., 2015; Bratman et al., 2015) and our results indicate that a natural setting alone is beneficial. In our study, combined DRMT/HMT and silence and a period of silence preceded by a group discussion on silence, both held in a city garden, significantly increased relaxation and affected the students’ perception of the duration of silence and the speed of time passage. Our findings coincide with the results from other studies (Kramer et al., 2013; Droit-Volet et al., 2015) and indicate an increased (more mindful) interoceptive awareness after meditation (Wittmann, 2015, 2018). Mindfulness is related to a positively experienced, slower passage of time. Participants in the DRMT/HMT setting who were more relaxed after the silence experienced a faster passage of time, suggesting that some individuals experienced states of “flow.” Time passes faster if one is fully immersed in activities (sports, work, performing music, playing) accompanied by positive feelings (Csikszentmihalyi and Csikszentmihalyi, 1988).

### “Pure” Silence (Study 5)

Study 5 (Pfeifer et al., 2019b) also involved students as participants and a 6:30 min period of silence of unknown duration to subjects. We did not combine the period of silence with a preceding element, such as DRMT/HMT or a seminar/group discussion. We incorporated 6:30 min of silence alone conducted either indoors (in a university seminar room similar to study 3) or outdoors in a natural setting (in the same place in the city garden as in study 5). Both conditions were guided by the same professional music therapist (E.P.).

Forty-six participants completed this study (42 women, 4 men; mean age: 23.5 years; *SD* = 4.9; range: 20–52). Subjects were regular students at the Catholic University of Applied Sciences Freiburg registered in the undergraduate BA program “Inclusive Education.” The participating students were divided into two groups and experienced the two conditions in reverse order with 1 week between each session, which began with a brief introduction focusing on general information (e.g., study aims, informed consent). Students then filled out questionnaires before and after the period of silence. They were also asked to take off wrist watches, to switch off and put away mobile devices, and to sit comfortably and stay seated and awake during the silent phase.

State scales on subjective time, self, and space (STSS) (Pfeifer et al., 2016), a VAS measuring relaxation, and a VAS measuring boredom related to the period of silence were used. We also included the Zimbardo Time Perspective Inventory (ZTPI) (Zimbardo and Boyd, 1999) and the Barratt Impulsiveness Scale (BIS-11) (Barratt et al., 1999).

The period of silence led to a significant increase in relaxation in both conditions (city garden and university seminar room). However, in the natural setting of the city garden, students experienced less boredom during the 6:30 min of silence than in the indoor condition. In the outdoor condition, the sense of being present was enhanced (experiencing the moment), while thoughts about the past (memories) were reduced. The more relaxed the students were after the silence indoors, the less intensely they were aware of time. There were hardly any significant correlations of the subscales of the ZTPI and the BIS-11 with any of the state variables. In fact, merely one significant correlation with one state variable was identified: the higher the negative past perspective, the lower the rating of the perceived self in the natural setting of the city garden.

Simply being surrounded by nature seems to positively affect psychological well-being by being more relaxed and feeling less boredom. Our findings coincide with empirical study outcomes emphasizing the relaxing, health-fostering, and recuperative effects of nature and natural surroundings (Ulrich, 1979; Berger and Lahad, 2013; Berry et al., 2015). Results also support the theory that nature can play an active role as a co-therapist in therapeutic processes (Pfeifer, 2017). Participants in the outdoor condition felt more in the here and now (present) and less in the past, which correlates with Jordan’s (2015) claim that working outdoors with patients during therapy increases their present awareness and “now moments.” Previous investigations showed negative correlations between dispositional mindfulness – an increased ability to be present-oriented – and the propensity of mind-wandering (Mrazek et al., 2012; Weiner et al., 2016). Mindfulness meditation decreases mind-wandering measured as less distraction from task-related performance (Mrazek et al., 2012; Mooneyham and Schooler, 2013). Our study results indicate that simply being exposed to silence in a more natural setting has an effect similar to those experienced in mindfulness interventions. Exposure to nature reduces rumination and associated prefrontal-cortex activation (Bratman et al., 2015). Whereas preceding studies indicated that meditative music is more relaxing if intermingled with silence (Bernardi et al., 2006) or that silence has significant relaxing effects if combined with DRMT/HMT (Pfeifer et al., 2016), this study indicates that silence itself experienced in a group in a natural setting is beneficial, especially in the presence of a qualified therapist (Grawe et al., 1997).

## Concluding Discussion of the Main Results

“After all, silence is not nothing. It is better to say that from something comes something” (Kagge, 2018). We agree, as several minutes of silence affects time perception and mood, leads to more relaxation, and changes the perception of self and space. The following sections (see also Table 1) subsume relevant findings of our studies and discuss health-related therapeutic and educational contexts. Our use of the term silence includes the various “silent situations” in our studies (a period of “just thinking,” a real waiting situation, “pure” silence in a natural setting, a period of silence following DRMT/HMT or following a group discussion as part of a seminar). We relied on relative rather than absolute or total silences in all of our studies.

**TABLE 1:** An overview of the main results of our studies regarding the students’ experience of a period of silence in varying conditions.

	**Study 1 (Waiting room)**	**Study 2 (“Just think”)**	**Study 3 (DRMT indoors)**	**Study 4 (DRMT outdoors)**	**Study 5 (“pure” silence)**
Passage of time	Judged to be fast when relaxed and in a positive mood	Judged to pass quickly		Judged to be slower in DRMT than in the seminar condition (71% of subjects)	
Duration of time	Underestimated when relaxed and in a positive mood		Overestimated in DRMT vs. the seminar condition (76% of subjects)	Overestimated in DRMT vs. the seminar condition (71% of subjects)	
Intensity of time			Felt less intensely in DRMT vs. the seminar condition (58% of subjects)		
Time orientation		Increased present orientation	Reduced future perspective in the DRMT condition (51% of subjects)		Increased present orientation and reduced past perspective outside (76% of subjects)
Relaxation		Increased	Increased; greater increase in DRMT	Increased in both conditions (DRMT, seminar)	Increased; greater increase outdoors
Mood and emotion	Correlation with subjective time	Less aroused and in better mood			
Perception of space			Decreased in DRMT vs. seminar		
Perception of self		High; more self-awareness			
Impulsiveness and mindfulness	Correlation with subjective time and relaxation	Did not have an impact on results	Did not have an impact on results		Did not have an impact on results
Boredom		Hardly felt		Boredom leads to lower relaxation effects	Felt less outdoors vs. indoors

### Time Perception and Time Orientation

In the waiting-room study (study 1), those students who were more relaxed and judged the passage of time to have elapsed more rapidly also estimated the duration for the waiting period to be comparably shorter than individuals who were more irritated because of having to wait for an uncertain period of time. When subjects were instructed to “just think,” time on average was judged to pass comparably quickly, although the subjects were alone in the room (study 2). During silence in a natural setting after the DRMT/HMT intervention, participants experienced time to have passed significantly slower than during a period of silence preceded by a seminar group discussion in the same natural surrounding (study 4). This is the specific effect of the music-therapy, depth-relaxation intervention similar to a mindfulness intervention. Participants significantly overestimated the duration of the period of silence after DRMT/HMT both indoors and outdoors (studies 3 and 4).

Mind-body interventions, such as meditation, yoga, and other relaxation techniques, like resting in a floatation tank, have been introduced into the clinical and health sciences (Esch et al., 2013; Feinstein et al., 2018; Kohls et al., 2019). Combined DRMT/HMT and silence could be an effective approach to induce relaxation. Short breaks of silence could prove beneficial when taken regularly to lessen the pressure of modern life (Berger and Lahad, 2013) caused by noise (pollution), stress, time constraints, and other factors (Lederbogen et al., 2011; German Federal Ministry of Education and Research, 2016; Lederbogen and Meyer-Lindenberg, 2016; Kagge, 2018).

A period of silence also altered the participants’ subjective time orientation. The present orientation (experiencing the moment) increased during silence, and the future perspective (plans and expectations) and the past perspective (memories) decreased (see studies 2, 3, 5). This finding is important from a therapeutic and educational point of view. Rumination, the negative form of being engaged with one’s own thoughts about the past or the future, can enhance passivity and prevent one from being active and effective in the present (Frankl, 2006, 2008; Markert, 2018). Rumination is also associated with depression, anxiety, and obsessive-compulsive disorders (Modini and Abbott, 2016; Lawrence et al., 2018; Tibi et al., 2018; Nasiri et al., 2019). Silence in a natural setting or indoors and/or combined with DRMT/HMT may be comparable to mindfulness-based approaches’ positive outcomes in reducing rumination and mind wandering (Frostadottir and Dorjee, 2019; Gutiérrez et al., 2019) and foster a present orientation. In Frankl’s humanistic approach in psychotherapy (logotherapy), such a focus on the present moment is crucial to realize meaning in our lives. “Meaning also rests in the appreciation of the moment. When our awareness is focused on the past or on the future, we lose connection to *now*. […] When we work in awareness of the moment, we stay connected to meaning” (Pattakos and Dundon, 2017). *Dereflection* is a method applied in logotherapy to help move one’s attention away from negative (rumination) to more positive and enjoyable thoughts and situations (Frankl, 2014). Silence could be a useful “tool” to accompany methods like *dereflection* in the treatment of depression, anxiety, and obsessive-compulsive disorders.

### Relaxation

Studies two to five showed a significant increase in participants’ relaxation after vs. before the silent interval. Regardless of whether students experienced silence alone in a room (just thinking), as part of a group indoors or outdoors in nature, or combined with DRMT/HMT, they felt on average significantly more relaxed after silence. Silence as part of the experimental conditions in studies two (“just think”), three (DRMT indoors), four (DRMT outdoors), and five (“pure” silence in nature) led to significantly greater relaxation.

Surveys highlight the fact that stress is a common reaction among students these days (AOK Bundesverband, 2016). Participants in our studies were students who judged silence to be effective in increasing relaxation. Implementing silence as a relaxation-inducing practice into educational settings, applied indoors, in nature, or preceded by DRMT/HMT, could enhance students’ and teachers’ well-being. Experiencing silence from time to time could help prevent stress-related diseases and reactions like burnout. Previous investigations in educational settings using MBSR-interventions showed positive results in this context (Gouda et al., 2016). Silence is a flexible and an effective resource to enhance relaxation. Our findings add to knowledge regarding popular relaxation techniques, such as mindfulness meditation, progressive muscle relaxation, autogenic training, guided imagery, and hypnotherapy. The combination of silence and DRMT/HMT as an effective approach widens the spectrum of music-centered relaxation techniques (Frohne-Hagemann, 2007; Stegemann, 2013). Further research on the implementation of silence and DRMT/HMT in different therapeutic and educational settings is indicated.

### Mood and Emotion

In the real-waiting-time study (study 1), students with more positive emotions and higher scores in relaxation felt that time had passed faster during the silent waiting situation and relatively underestimated its duration. Conversely, the period of waiting was over-estimated when subjects felt more irritated. Subjective time and mood are interrelated. When asked to wait and “just think” (study 2), students felt significantly less aroused and in a better mood after a period of silence alone in a room. These reactions are typical signs of relaxation and flow induced through various activities (Csikszentmihalyi and Csikszentmihalyi, 1988; Conti, 2001; Pfeifer et al., 2016; Wittmann, 2016). Being alone in a room for a period of silence in the range of a few minutes positively affected emotion, arousal, and relaxation in a similar way. Nguyen et al. (2017) conclude that solitude could induce affective self-regulation. Such an approach could be useful in clinical and non-clinical therapeutic and educational settings fostering well-being and health.

### Perception of Space and Self

A period of silence preceded by a session of DRMT/HMT led to a significant decrease in the perception of space (study 4). This coincides with outcomes linked to comparable induction techniques, such as rhythm-induced trance, meditation, or listening to music. These lead to alterations in states of consciousness and mutually affect the senses of self, time, and space (Berkovich-Ohana et al., 2013; Schäfer et al., 2013; Wittmann, 2015).

The sense of self was comparably intense when just thinking in silence alone in a room (study 2; an average of 5 with an item range between 0 and 6). An increased sense of self is often associated with boredom and irritation combined with a slow passage of time (Deinzer et al., 2017). In our study, the “simple” task of engaging oneself with one’s own thoughts in silence led to greater self-awareness in an emotionally positive way (study 2; Pfeifer et al., 2019c). From a therapeutic perspective, silence “[…] may be a fertile space to refocus and redirect creativity or a space of self-reflection […]” (Kirkland, 2013). In her meta-analysis, Pesek (2007) concluded that music therapy is highly effective (0.83; according to Cohen’s d) in supporting patients’ self-concepts. Accordingly, silence could, on the one hand, be a meaningful addition to the methodical approaches in music therapy, and, on the other hand, a promising topic for future research initiatives.

### Impulsiveness, Mindfulness, Boredom

Whereas the participants’ self-rated impulsivity affected relaxation and time estimation during a silent period of waiting (study 1), the students’ individual levels of impulsiveness and mindfulness did not have an impact on the results of studies two, three, and five. Impulsiveness and mindfulness did not interfere with the positive results (increased relaxation, better mood, less arousal, stronger present orientation) resulting from a period of silence spent in nature, being engaged with one’s own thoughts or as part of a DRMT/HMT session. This negative finding may be due to the group of studied subjects who were mainly students. In this comparably homogeneous group of individuals, the variance of traits was probably limited. Considering this, silence as used in clinical and therapeutic applications will have to be investigated furthermore with different patient groups in order to see how traits such as impulsiveness might affect DRMT/HMT interventions.

Students hardly felt boredom during 6:30 min of just thinking in silence alone in a room (study 2) and experienced significantly less boredom during 6:30 min of silence in nature than during a silence of equal length in a seminar room at a university (study 5). Correlations could be found in study 4. The more bored individuals were before the outdoor group discussion/seminar condition, the less relaxed they were after the period of silence. In the DRMT/HMT condition, those participants who had significantly higher scores on the boredom-arousal scale were less relaxed afterward. Higher pre-intervention levels of boredom in participants seem to lower the relaxing effects of a period of silence. Frankl (1992; Pattakos and Dundon, 2017) correlated boredom with what he called the “existential vacuum.” He claimed that boredom leads to more problems than distress and referred to phenomena such as depression, aggression, addiction, and even suicide in his discussion of problems resulting from boredom and an existential vacuum. Although further research needs to be conducted, our findings, in correlation with Frankl’s assumptions, suggest that silence is effective in many ways, with boredom hardly being perceived during silence. Boredom was lower in nature as compared to the indoors situation. Nature itself seems to be effective regarding the lack of boredom. Nature experiences reduce rumination and associated prefrontal-cortex activation (as one study showed: Bratman et al., 2015). This could provide a meaningful approach to the prevention and therapeutic treatment of diseases/symptoms like depression, addiction, and aggression.

### Nature, the Therapeutic Relationship, and DRMT/HMT as Influencing Factors

In studies 1 and 2, participants experienced silence alone in a room while just thinking or waiting. The situations in studies 3–5 were held outdoors, and the subjects took part in a group in the presence of a music therapist. According to Grawe et al. (1997), the therapeutic relationship is one of the five effect factors in psychotherapy. A music therapist is specifically qualified to prepare and lead individuals through acoustic experiences, including silence, providing an atmosphere of reliability (Petersen, 1996). These factors were also crucial to the performance of DRMT/HMT. Although our studies were not performed in a clinical or therapeutic context, DRMT/HMT is a music-therapeutic method that only qualified therapists should apply. Even outside a professional DRMT/HMT setting, silence in nature had a positive effect by inducing relaxation.

In study 4, one of the conditions took place outdoors in a city garden. In study 5, both conditions were performed in the city garden. The influence of nature was the focus of these studies. Pfeifer (2017) once described nature as a co-therapist in therapeutic processes. Simply being surrounded by nature in silence positively affects psychological well-being, promotes relaxation, decreases boredom, and increases the present orientation. Previous investigations led to similar results (Ulrich, 1979, 1984; Berger, 2009; Berger and Lahad, 2013; Berry et al., 2015; Bratman et al., 2015; Davydenko and Peetz, 2017). Jordan (2015) found “a growing evidence base that points toward the role of nature and its preventative and curative effects […] and [a]n increasing number of therapists […] taking their practices outdoors and walking with their clients while conducting therapy.” A study by Lee et al. (2015) showed that green micro-breaks consisting of a 40 seconds view of a flowering meadow on a green roof boost multiple attention networks. Our findings provide further insights that encourage this outdoor-therapy tendency. Combined silence and nature may also prove useful to work settings and relevant psychological requirements.

## Perspectives

Silence, as part of waiting or “just thinking” situations, combined with DRMT/HMT, alone or in a group setting, indoors or outdoors, is effective in many ways. A few minutes of silence significantly increased relaxation, improved mood states, altered the perception of time and self, and the orientation toward the present moment.

Statements by Antonovsky (1979); Becker (2000), Frankl (1992, 2014), and Maslow (1968, 1987) highlight the importance of meaning in life to health and well-being. Why not interlink silence and meaning in life in subsequent studies? Only few attempts have been made in this direction so far. Alahmadi et al. (2017), for example, mention that a silent period of thinking for pleasure can be a viable way to meet people’s goal to find meaning. According to Frankl (1992), one can discover meaning by realizing creative values, experiential values, or attitudinal values. He lists arts and music as relevant to these values (Frankl, 2004). Current arts-based conceptualizations in healthcare (e.g., *music, health, and well-being*) define music through its social function. Depending on the social context, silence, like any sound, may be labeled music (MacDonald et al., 2012). Cage (1973) argued similarly: “The material of music is sound and silence.” Therefore, silence could possibly enhance meaning related to all three value categories: experiencing silence as part of a relaxation or mindfulness exercise (experiential values), silence involved in a creative process (creative values; e.g., a musical work, such as 4′33″ by John Cage), and silence as part of a therapeutic process leading to an attitude change toward unavoidable suffering (attitudinal value) (Pfeifer, 2019).

Pattakos and Dundon (2017) identified a growing lack of meaning among employees in the working world. A lack of meaning can negatively affect well-being, performance, resilience, work quality, and engagement, as regularly demonstrated in Gallup polls (Gallup, 2017). Further investigations could investigate the benefits of silence in work settings.

Our studies were of quantitative nature. Our study designs led to a variety of results and insights regarding silence in different situations, surroundings, and settings. Qualitative approaches would definitely provide further insights into the subjective perception of silence. Silence is a multifaceted phenomenon, and our studies indicate its potentials for therapy, education, and the working world.

## Author’s Note

In memory of our dear friend and colleague Henrike Fiedler (1991–2019).

## Author Contributions

The empirical work reported in this review was designed, planned, and statistically evaluated by EP and MW as the principal investigators. EP drafted and revised the manuscript. MW provided substantive suggestions for revisions and critically reviewed the manuscript. Both authors reviewed and approved the final manuscript.

## Conflict of Interest

The authors declare that the research was conducted in the absence of any commercial or financial relationships that could be construed as a potential conflict of interest.
